# Validity of the Japanese Orthopaedic Association Hip Disease Evaluation Questionnaire (JHEQ) for Japanese patients with labral tear

**DOI:** 10.1093/jhps/hnaa038

**Published:** 2020-10-29

**Authors:** Nobuyuki Watanabe, Satona Murakami, Soshi Uchida, Satoshi Tateishi, Hidetsugu Ohara, Yasuhiro Yamamoto, Taiki Kojima, JAHORN (Japan Arthroscopy of the Hip Outcomes Research Network)

**Affiliations:** 1 Department of Orthopaedic Surgery, Tosei General Hospital, Seto City, Aichi 489-0065, Japan; 2 Department of Orthopaedic Surgery, Graduate School of Medical Sciences, Nagoya City University, Nagoya City, Aichi 467-8602, Japan; 3 Department of Rehabilitation Medicine, Graduate School of Medical Sciences, Nagoya City University, Nagoya City, Aichi 467-8602, Japan; 4 Department of Orthopaedic Surgery, Wakamatsu Hospital of the University of Occupational and Environmental Health, Kitakyusyu City, Fukuoka 808-0024, Japan; 5 Department of Rehabilitation Medicine, Wakamatsu Hospital of the University of Occupational and Environmental Health, Kitakyusyu City, Fukuoka 808-0024, Japan; 6 Department of Orthopaedic Surgery, Hirakata City Hospital, Hirakata City, Osaka 573-1013, Japan; 7 Department of Occupational Therapy, Health Science University, Minamitsuru-gun, Yamanashi 401-0380, Japan; 8 Department of Anesthesiology, Aichi Children's Health and Medical Center, Cincinnati, Ohbu City, Aichi 474-8710, Japan

## Abstract

The Japanese Orthopaedic Association Hip Disease Evaluation Questionnaire (JHEQ) was created for patient-reported outcome measures (PROMs) and to evaluate the conditions of patients with hip disease. Nevertheless, the validity of the JHEQ for patients with hip labral tears remains unclear. Therefore, we validated the JHEQ in patients with labral tears. There were 51 patients (mean age 44.5, range 18–60 years; 31 women). Thirty-two patients had right-sided tears, 29 underwent hip arthroscopy, 32 had femoroacetabular impingement and 15 had developmental hip dysplasia. Five PROMs included in the JHEQ were evaluated using test–retest methods. Statistical analysis was performed using SPSS software according to the COnsensus-based Standards for the selection of health status Measurement Instruments checklist. The intra-class correlation coefficient (1, 2) of all JHEQ scores (84 points) was 0.88 and Cronbach’s *α* was 0.94. Bland–Altman analysis revealed good test–retest reliability for the JHEQ. The Spearman’s rank test, including the SF-36 subscale, showed a high correlation with physical functioning [1, 0.67 (*P* < 0.01); 2, 0.65 (*P* < 0.01)], body pain [1, 0.54 (*P* < 0.01); 2, 0.53 (*P* < 0.01)] and physical component summary [1, 0.55 (*P* < 0.01); 2, 0.55 (*P* < 0.01)]. The value of minimal important change (22.9) was higher than that of smallest detectable change (3.21), suggesting that the JHEQ has adequate responsiveness. We demonstrated the reliability, validity and responsiveness of the JHEQ in Japanese patients with hip labral tears. JHEQ is a valid assessment tool not only for patients with hip osteoarthritis or osteonecrosis but also for those with hip labral tears.

## INTRODUCTION

Patient-reported outcome measures (PROMs) are important metrics for assessing therapeutic effects because of the possible discrepancies among therapists and patients with respect to clinical evaluations. PROMs include the perception of rheumatological disease activity [1], post-operative pain following hip surgery, degree of focus on social rehabilitation and influence of therapists’ bias on clinical outcomes evaluations [2]. The Japanese Orthopaedic Association Hip Disease Evaluation Questionnaire (JHEQ) was developed using data from patients with osteoarthritis (75.1%) and osteonecrosis (13.9%) [3]. Nevertheless, the validity of the JHEQ for patients with hip labral tears is unclear.

The number of arthroscopic hip surgeries for acetabular labral tears and femoroacetabular impingement (FAI) has been increasing worldwide [4–6]. PROMs, such as the Non-Arthritis Hip Score [7] and the Hip Outcome Scores [8], have been accepted as useful clinical measures [9–11]. However, these PROMs were developed in English-speaking regions only; therefore, direct application of PROMs in the English language in patients who do not speak English as their first language may not be optimal because of the potential misunderstanding and lack of comprehension regarding the content of the inquiries. Currently, only two internationally validated arthroscopic hip surgery PROMs are available in Japanese: the Japanese version of the Oxford Hip Score (OHS) [12, 13] and the JHEQ [3]. The OHS was developed to evaluate artificial hip joint replacement [12, 13] and the subjects have physical restrictions because of hip pain (e.g. hip pain restricts walking and performing housework). Because the OHS included only patients with hip pathologies, the scale could not be applied to highly active patients (e.g. athletes) who undergo arthroscopic hip surgery [12, 13]. We developed a Japanese version of the International Hip Outcome Tools 12 (iHOT12J) and the Vail Hip Score (Vail10J) [14, 15]. During the development of the Japanese version of these PROMs, we also performed a test–retest analysis for the JHEQ and evaluated the validity of JHEQ for patients with hip labrum tears.

## MATERIALS AND METHODS

This study was approved by our hospital ethics committee. For the development of the JHEQ by Matsumoto *et al.*, 12 university hospitals and 5 municipal hospitals were included. The first 464 comments were obtained from ∼100 patients using open question methods. Based on the comments and stocked items, 58 question items were formulated. These questionnaires included 402 patients (75.2% had osteoarthritis and 15.3% had osteonecrosis). In view of the results of analysis of these categories, they selected 21 question items, in consultation with clinicians, regarding each factor and considered the naming of the categories (pain, movement and mental) [3].

When the JHEQ is administered simultaneously with other PROMs such as the short form-36 (SF-36), there is no significant increase in the number of question items. We examined the reliability, validity and responsiveness of the JHEQ according to the Consensus-based Standards for the selection of health status Measurement Instruments (COSMIN) checklist [16]. The COSMIN checklist is a tool to assess the methodological quality of studies on measurement properties of health status measurement instruments based on an international Delphi consensus.

### Methodological testing according to the COSMIN checklist with grading system

The COSMIN checklist has five grading levels: very good, adequate, doubtful, inadequate and not applicable. In this study, there were 10 boxes for the evaluation of PROMs development, content validity, structural validity, internal consistency, cross-cultural validity/measurement invariance, reliability, measurement error, criterion validity, hypotheses testing for construct validity and responsiveness. All boxes were graded according to the five-point scale. The evaluated items were graded as ‘very good’ or ‘adequate’. The grades were identified as adequate using statistical analyses, missing item handling and data collection methods [17].

### Data collection

Data regarding PROMs were obtained between March 2016 and October 2017 from three community hospitals (Facility A, Facility B, Facility C) and one university hospital (Facility D) in Japan. The inclusion criteria included a diagnosis of acetabular labral tears based on an imaging modality, age between 18 and 60 years old and activity levels >3 based on Tegner Activity Score [18]. The exclusion criteria were analogous to those of the iHOT33 (e.g. polytrauma and active joint infection) [19]. To avoid the risk of bias, physicians did not obtain data. In the second PROMs data collection, we provided pre-stamped envelopes for the patients’ convenience. We conducted the assessments with an identical set of PROMs (five measures: JHEQ, iHOT12J, Vail10J, Japanese version of the OHS and SF-36) twice within 6 months at 2-week intervals in patients who were experiencing the therapeutic effects of surgery for >3 months and who were receiving conservative treatment. The JHEQ has a separate visual analogue scale (VAS) for patient satisfaction (lowest 100, highest 0) in addition to its scored items; the questionnaire was composed of 15 subsections and 93 items. The iHOT12J consists of 12 items, and all items adopted a 100 mm VAS format. For SF-36, we used the Japanese version (SF-36 ver. 2) [20]. The IRB of each facility also provided approval. Written informed consent was collected from all patients who agreed to enrol in this study.

### Reliability

We evaluated reliability, test–retest reliability, internal consistency and measurement error [21]. Intra-class correlation coefficient (ICC) was calculated to assess the test–retest reliability; values >0.7 was considered sufficient to support test–retest reliability [21]. Internal consistency was measured by Cronbach’s alpha for each subscale of the JHEQ. The values between 0.70 and 0.95 were considered to indicate good internal consistency [21]. Bland–Altman analysis and limit-of-agreement calculation were performed to assess the absolute agreement between the first and second tests of the JHEQ [22]. The average difference between the first and second tests of all JHEQ subscales with 95% confidence interval (CI) was determined and 95% of the limits of agreement was calculated using the following formula: average difference ± 1.96 × the standard deviation (SD) of the mean difference between the first and second tests (SD_diff_).

### Validity

To test validity, we chose the SF-36, which has national generalized data. For SF-36 and reproducibility of the JHEQ, we adopted the established Japanese version of the SF-36 and investigated three components: physical functioning (PF), body pain (BP) and physical component summary (PCS). For discrimination validity, we examined the relationships between the following five components, which are subscales of SF-36 measuring psychological aspects: general health [20], vitality, social functioning (SF), emotional role functioning (role-emotional, RE) and mental health (MH). We also analysed the three quality of life (QOL) summary scores (PCS; mental component summary (MCS) and role/social component summary (RCS)] [23]. For JHEQ data collection, we utilized the actual VAS data (both for satisfaction VAS and pain VAS) plotted on 100 mm lines on paper (0–100). For the SF-36, the analysis was performed by inputting response numbers into an Excel sheet with an automatic calculation formula. Spearman’s rank correlation coefficients were evaluated to identify the correlations among the JHEQ, iHOT12J, OHS, Vail10J, satisfaction VAS, eight subscales of SF-36 and three QOL summary scores.

### Responsiveness

Responsiveness is the ability of a questionnaire to detect a change in the construct measured over time [17] and is assessed by comparing the smallest detectable change (SDC) with the minimal important change (MIC) [21]. SD is a statistical measure and reflects the smallest change in the scores of each subject that can be considered a real change [22]. In this study, standard error of measurement was calculated using the formula SD/√(1-ICC) (ICC, intra-class correlation coefficient) and SDC was calculated using the formula $\mathrm{SEM}\;\times \;1.96\times \sqrt{2}/\sqrt{n}$. Responsiveness was interpreted as sufficient if the SDC < MIC [24]. Additionally, an anchor-based method was applied to evaluate responsiveness [25].

### Sample size

A sample size of 50 is required to assess the validity of JHEQ according to the COSMIN checklist. The sample size in this study (*n* = 51) was evaluated as ‘adequate’ according to the COSMIN checklist [17]. All statistical analyses were performed using SPSS version 25 statistical software (IBM Corp., Armonk, NY, USA). A *P* value <0.05 was considered statistically significant.

## RESULTS

From the four facilities, we enrolled a total of 72 patients and 73 hip joints. After excluding data with entry errors or omissions, usable data were obtained from 50 patients and 51 joints (70%). The patient distribution was as follows: 14 patients were from Facility A (28%), 10 from Facility B (20%), 14 from Facility C (28%) and 12 from Facility D (24%). For disorders, 32 patients had FAI (64%), 15 had developmental dysplasia of the hip (30%) and 3 had other disorders (6%). The average age was 44 years (range 18–60); 31 were women (61%). There were 33 right-sided hip injuries (65%) and 18 left-sided injuries (35%). The treatment included arthroscopic surgery in 29 cases (57%) and conservative therapy in 22 cases (43%) (Table I).

**Table I.** Patient characteristics (n = 51)

Characteristics	Mean ± SD (range) or *n* (%)
Age (years)	
Mean	44
Range	18–60
Sex	
Women	31 (61)
Men	20 (39)
Side	
Right	33 (65)
Left	18 (35)
Treatment	
Hip arthroscopy	29 (57)
Conservative	22 (43)
Diagnosis	
FAI	32 (64)
DDH	15 (30)
Others	3 (6)

SD, standard deviation; FAI, femoroacetabular impingement; DDH, developmental dysplasia of the hip.

### Reliability

For the data collected for VAS and subgroup of the JHEQ (Table II), we determined the ICC for the first and second tests (1, 2) and the Cronbach’s *α* coefficient. The ICC for the average of all JHEQ scores was 0.88 and the Cronbach’s *α* coefficient was 0.94 (Table III).

**Table II.** Change in JHEQ score

	First round				Second round			
JHEQ subgroup	Mean ± SD	Floor effect (%)	Ceiling effect (%)	Median (IQR)	Mean ± SD	Floor effect (%)	Ceiling effect (%)	Median (IQR)
Satisfaction VAS	40.4 ± 31.6	2	4	25 (100–0)	36.5 ± 30.4	6	2	24 (100–0)
Pain VAS	18.5 ± 23.3	0	0	7 (87–1)	16.7 ± 23.3	0	2	6 (83–0)
Pain	19.0 ± 6.3	0	5.9	19 (6–28)	19.5 ±5.8	0	7.8	19 (8–28)
Movement	18.8 ± 7.3	0	15.7	20 (1–28)	19.8 ± 6.4	0	15.7	21 (6–28)
Mental	20.4 ± 6.3	0	15.7	21 (4–28)	20.4 ± 6.3	0	17.6	20 (9–28)
JHEQ	58.2 ± 16.9	0	2.0	60 (21–84)	59.8 ± 15.5	0	3.9	59 (30–84)

SD, standard deviation; IQR, interquartile range; JHEQ, Japanese Orthopaedic Association Hip Disease Evaluation Questionnaire.

**Table III.** Intra-class correlation coefficients in the JHEQ subgroups

Subgroups JHEQ^a^	ICC	95% CI	Cronbach’s *α*
Satisfaction VAS	0.52	0.29–0.69	0.68
Pain VAS	0.94	0.89–0.96	0.97
Pain point from VAS	0.92	0.87–0.96	0.96
Pain categories	0.85	0.75–0.91	0.92
Movement categories	0.89	0.81–0.93	0.94
Mental categories	0.80	0.67–0.88	0.89
Total score JHEQ^b^	0.88	0.79–0.93	0.94

ICC, intra-class correlation coefficients; CI, confidence interval.

a JHEQ has three subgroups (pain, movement and mental) and two VAS (satisfaction VAS and pain VAS).

b The total score of JHEQ is the average of added 28th Questioner scores (0–4 points).

**Fig. 1.**
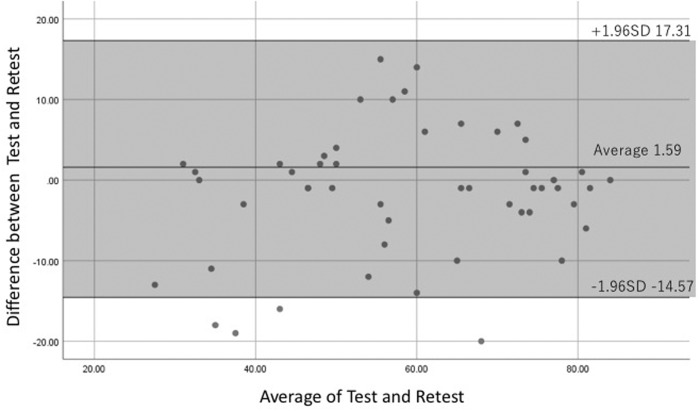
Bland–Altman plot for test–retest reliability of the Japanese Orthopaedic Association Hip Disease Evaluation Questionnaire. Each data point indicates the difference between the test and retest for an individual patient. Grey area shows the 95% (±1.96 SD) limits of agreement.

Bland–Altman analysis showed a difference of zero, lying within the 95% CI, between the first and second tests of JHEQ, thereby ruling out systematic bias (Fig. 1). The test–retest reliability of JHEQ was 1.59 on average (Fig. 1). The limits of agreement were from −14.6 to 17.3 on the Bland–Altman plot (Fig. 1). Bland–Altman plot for test–retest reliability of the JHEQ shown each data point indicated how the difference between test and retest for individual patient compares with the mean of the two sessions for scores of JHEQ.

### Validity

Spearman’s rank correlation coefficient was *r* > 0.50 (*P* < 0.01) for both the first and second assessments of the JHEQ, with iHOT12J, Vail10J, JHEQ, satisfaction VAS and SF-36 subscales (PF, BP and PCS, Table IV). A relatively high correlation with PF (first, *r* = 0.67, *P* < 0.01; second, *r* = 0.65, *P* < 0.01) and PCS (first, *r* = 0.55, *P* < 0.01; second, *r* = 0.55, *P* < 0.01) was found. By contrast, the correlations were weak with the respect to following components: SF (first, *r* = 0.32, *P* < 0.05; second, *r* = 0.43, *P* < 0.01), RE (first, *r* = 0.18, *P* > 0.05; second, *r* = 0.43, *P* < 0.01), MCS (first, *r* = 0.34, *P* > 0.05, *P* < 0.01; second, *r* = 0.30, *P* < 0.05) and RCS (first, *r* = 0.08, *P* > 0.05; second, *r* = 0.14, *P* > 0.05, Table IV).

**Table IV.** Spearman’s rank correlation coefficients in the JHEQ

Domain/subdomain	Average of the first round (SD)	Correlation of the first round	Average of the second round (SD)	Correlation of the second round
OHS (0–48)	35.7 (11.6)	0.30^*^	36.5 (12.2)	0.25
iHOT12J (0–100)	63.1 (24.8)	0.80^**^	68.6 (24.2)	0.82^**^
Vail10J (0–100) ^a^	71.6 (16.5)	0.79^**^	74.4 (16.3)	0.86^**^
JHEQ satisfaction VAS (100–0)	40.4 (31.6)	−0.55^**^	36.5 (30.4)	−0.66^**^
SF-36				
Physical functioning	80.3 (18.8)	0.67^**^	81.7 (17.8)	0.65^**^
Role—physical	81.4 (24.8)	0.37^**^	84.3 (22.1)	0.43^**^
Bodily pain	67.7 (16.7)	0.54^**^	71.0 (17.9)	0.53^**^
General health	63.6 (18.1)	0.47^**^	63.9 (16.9)	0.50^**^
Vitality	60.6 (21.9)	0.40^**^	63.6 (21.6)	0.45^**^
Social functioning	88.2 (18.9)	0.32^*^	90.9 (15.2)	0.43^**^
Role—emotional	86.1 (21.8)	0.18	88.9 (18.3)	0.43^**^
Mental health	71 (19.9)	0.46^**^	76.8 (17.8)	0.34^*^
Physical component summary	43.9 (11.2)	0.55^**^	43.8 (11.4)	0.55^**^
Mental component summary	51.7 (9.8)	0.34^*^	53.3 (9.3)	0.30^*^
Role/social component summary	49.6 (12.1)	0.08	51.4 (10.9)	0.14

SD, standard deviation; OHS, Oxford Hip Score; iHOT12J, international Hip Outcome Score 12 Japanese version; Vail10J, Vail Hip Score (Japanese version); SF-36, short form-36.

a Vail10J has four empty data on the first and second rounds.

* *P* < 0.05.

** *P* < 0.01.

### Responsiveness

The value of MIC (22.9) was higher than that of SDC (3.21); thus, the responsiveness of the JHEQ was considerate adequate. No relevant floor effect for the JHEQ was noted as no patient had a score of zero. Only two patients (3.9%) scored the maximum score of 84, which was the reason of the reduction in relevant ceiling effect (Table II).

## DISCUSSION

Seki *et al.* [26] validated the JHEQ with 82 patients with hip osteoarthritis and necrosis using test–retest methods and reported excellent reliability (ICC > 0.85); however, no validated study for patients with hip labral tears has been conducted. This is the first multi-institutional descriptive study that assessed the validity of the JHEQ in patients with acetabular labral tears. We found that the reliability, validity and responsiveness of the JHEQ were satisfactory and that the JHEQ could be used as a valid and reliable measure in patients with hip labral tear and FAI.

Overall, the assessment of the JHEQ yielded remarkably high values, with an ICC of 0.88 and a Cronbach’s *α* coefficient of 0.94. The ICC for each question item in both rounds of the JHEQ assessment ranged from 0.80 to 0.94, except that of satisfaction VAS. Cronbach’s *α* coefficients ranged from 0.68 to 0.97, demonstrating high reproducibility and reliability. Satisfaction VAS assesses patient satisfaction. Our study included patients with hip arthroscopy (57%) and those who received conservative treatment (43%), which suggests that patient background might affect the result of VAS. Emara *et al.* [27] followed patients who underwent conservative treatment over a 2-year period and reported that the patients tended to avoid activities that caused such symptoms, whereas those who had an operation tended to return to their sports activity. This suggests that the satisfaction between operated patients and those who had conservative treatment differs.

In the JHEQ and the Japanese version of the SF-36, strong correlations were identified in the subscales of physical components, specifically, PF, BP and PCS. By contrast, the correlations were weak with respect to the psychological components (SF, RE, MCS and RCS). Hence, the measures are minimally influenced by psychological components and are fully sufficient as physical assessment indices for hip joint disorders. In the subgroup analysis, JHEQ for pain showed a strong correlation with BP, and JHEQ movement had a strong correlation with PF and PCS. These subgroups have a strong correlation with adequate SF-36 categories. Moreover, JHEQ mental had a weak correlation with MH and MCS. Thus, JHEQ mental may not be relevant to the mental categories of the SF-36 in this study (Table V). Comparing the JHEQ with its satisfaction VAS, iHOT12J, Vail10J and Japanese version of the OHS, the correlations were weak with the OHS (first, *r* = 0.30, *P* < 0.01; second, 0.25, *P* > 0.05), whereas correlations were strong with the iHOT12J (first, *r* = 0.80, *P* < 0.01; second, *r* = 0.82, *P* < 0.01), Vail10J (first, *r* = 0.79, *P* < 0.01; second, 0.86, *P* < 0.01) and satisfaction VAS (first, *r* = −0.55, *P* < 0.01; second, *r* = −0.66, *P* < 0.01). The results of the OHS were possibly influenced by the fact that this measure was developed for advanced osteoarthritis of the hip requiring artificial joint replacement. Furthermore, the strong correlation with iHOT12J appears to have high cross-cultural reproducibility and validity in terms of patient backgrounds, language and culture [14].

**Table V.** Spearman’s rank correlation coefficients in the JHEQ subgroups

Domain	Correlation (first round)			Correlation (second round)		
Subdomain	JHEQ pain	JHEQ movement	JHEQ mental	JHEQ pain	JHEQ movement	JHEQ mental
PF	0.47^**^	0.69^**^	0.50^**^	0.51^**^	0.71^**^	0.47^**^
RP	0.18	0.46^**^	0.20	0.24	0.41^**^	0.41^**^
BP	0.57^**^	0.46^**^	0.36^*^	0.54^**^	0.48^**^	0.36^*^
GH	0.25	0.47^**^	0.43^**^	0.31^*^	0.61^**^	0.43^**^
VT	0.20	0.41^**^	0.41^**^	0.28^*^	0.48^**^	0.43^**^
SF	0.40^**^	0.23	0.20	0.28^*^	0.44^**^	0.39^**^
RE	0.04	0.21	0.15	0.39^**^	0.33^*^	0.40^**^
MH	0.28^*^	0.43^**^	0.46^**^	0.18	0.31^*^	0.35^*^
PCS	0.39^**^	0.58^**^	0.35^*^	0.45^**^	0.62^**^	0.36^**^
MCS	0.20	0.31^*^	0.39^**^	0.16	0.37^**^	0.28^*^
RCS	0.07	0.10	−0.33	0.10	0.05	0.20

SD, standard deviation; PF, physical functioning; RP, role-physical; BP, bodily pain; GH, general health; VT, vitality; SF, social functioning; RE, role-emotional; MH, mental health; PCS, physical component summary; MCS, mental component summary; RCS, role/social component sum.

* *P* < 0.05.

** *P* < 0.01.

This study has a few major limitations. The sample size (*n* = 51) was relatively small. According to the COSMIN checklist [24], a sample size of >100 and 50–99 is excellent and good, respectively. In addition, previous studies argued that a sample size >50 may be sufficient for a sound statistical analysis in the assessment of the validity of PROMs [28, 29]. In another PROMs in this study, Vail10J had 47/51 complete data for statistical analysis; therefore, there was a possibility the result of the validity analysis in this study might differ from those of others [15]. Among the categories in the JHEQ, satisfaction VAS yielded a low ICC (0.52) and Cronbach’s *α* (0.68) values, whereas other question items had high ICC (0.80–0.94) and Cronbach’s *α* (0.89–0.97) values. Furthermore, the JHEQ with assessments based on the average value of all the question items also showed high ICC (0.88) and Cronbach’s *α* (0.94) values. These findings imply the reproducibility and reliability of the JHEQ.

Internationally validated PROMs have been translated to different languages and used cross-cultural methods [30–34]. Recently, we also developed the iHOT12J and Vail10J [14, 15]. In this study, the use of JHEQ, which is one of the PROMs developed in Japan, in patients with hip labral tear was validated. In the daily life of East Asian people, sitting on flat floors and using squatting toilets are common. Because JHEQ and its English version has some questions about these lifestyles, JHEQ will be adaptable for evaluation of hip labral tears in East Asian patients.

Further prospective studies are needed to assess the clinical significance of JHEQ in patients with FAI who underwent surgical or conservative treatment. Further studies are warranted to advance the clinical and therapeutic processes for orthopaedic patients, including those with labral tears, arthroscopic surgery recipients and those who are highly active or athletic. Doing so will improve the field of orthopaedics worldwide.

We evaluated JHEQ’s validity in patients with acetabular labral tears. The results demonstrate the utility of JHEQ and its potential for the assessment of highly active patients such as athletes with FAI. We believe that, in addition to iHOT12J and Vail10J, the JHEQ is a beneficial assessment tool for Japanese patients with FAI.
